# Frontal networks in adults with autism spectrum disorder

**DOI:** 10.1093/brain/awv351

**Published:** 2016-01-27

**Authors:** Marco Catani, Flavio Dell’Acqua, Sanja Budisavljevic, Henrietta Howells, Michel Thiebaut de Schotten, Seán Froudist-Walsh, Lucio D’Anna, Abigail Thompson, Stefano Sandrone, Edward T. Bullmore, John Suckling, Simon Baron-Cohen, Michael V. Lombardo, Sally J. Wheelwright, Bhismadev Chakrabarti, Meng-Chuan Lai, Amber N. V. Ruigrok, Alexander Leemans, Christine Ecker, MRC AIMS Consortium, Michael C. Craig, Declan G. M. Murphy

**Affiliations:** ^1^ 1 Sackler Institute for Translational Neurodevelopment, and Department of Forensic and Neurodevelopmental Sciences, Institute of Psychiatry, King’s College, London, UK; ^2^ 2 NatBrainLab, Centre for Neuroimaging Sciences, Institute of Psychiatry, King’s College, London, UK; ^3^ 3 Cambridgeshire and Peterborough NHS Foundation Trust; ^4^ 4 Brain Mapping Unit, Department of Psychiatry, University of Cambridge, UK; ^5^ 5 Autism Research Centre, Department of Psychiatry, University of Cambridge, UK; ^6^ 6 Department of Psychology and Center for Applied Neuroscience, University of Cyprus, Cyprus; ^7^ 7 Centre for Integrative Neuroscience and Neurodynamics, School of Psychology and Clinical Language Sciences, University of Reading, Reading, UK; ^8^ 8 Centre for Addiction and Mental Health and Department of Psychiatry, University of Toronto, Canada; ^9^ 9 Department of Psychiatry, National Taiwan University Hospital and College of Medicine, Taiwan; ^10^ 10 Image Sciences Institute, University Medical Center Utrecht, Utrecht, The Netherlands; ^11^ 11 National Autism Unit, South London and Maudsley NHS Foundation Trust, Bethlem Royal Hospital, Beckenham, UK

**Keywords:** autism spectrum disorder, diffusion tractography, frontal networks, language, arcuate fasciculus

## Abstract

It has been postulated that autism spectrum disorder is underpinned by an ‘atypical connectivity’ involving higher-order association brain regions. To test this hypothesis in a large cohort of adults with autism spectrum disorder we compared the white matter networks of 61 adult males with autism spectrum disorder and 61 neurotypical controls, using two complementary approaches to diffusion tensor magnetic resonance imaging. First, we applied tract-based spatial statistics, a ‘whole brain’ non-hypothesis driven method, to identify differences in white matter networks in adults with autism spectrum disorder. Following this we used a tract-specific analysis, based on tractography, to carry out a more detailed analysis of individual tracts identified by tract-based spatial statistics. Finally, within the autism spectrum disorder group, we studied the relationship between diffusion measures and autistic symptom severity. Tract-based spatial statistics revealed that autism spectrum disorder was associated with significantly reduced fractional anisotropy in regions that included frontal lobe pathways. Tractography analysis of these specific pathways showed increased mean and perpendicular diffusivity, and reduced number of streamlines in the anterior and long segments of the arcuate fasciculus, cingulum and uncinate—predominantly in the left hemisphere. Abnormalities were also evident in the anterior portions of the corpus callosum connecting left and right frontal lobes. The degree of microstructural alteration of the arcuate and uncinate fasciculi was associated with severity of symptoms in language and social reciprocity in childhood. Our results indicated that autism spectrum disorder is a developmental condition associated with abnormal connectivity of the frontal lobes. Furthermore our findings showed that male adults with autism spectrum disorder have regional differences in brain anatomy, which correlate with specific aspects of autistic symptoms. Overall these results suggest that autism spectrum disorder is a condition linked to aberrant developmental trajectories of the frontal networks that persist in adult life.

## Teaser

Autism spectrum disorder (ASD) is proposed to reflect atypical connectivity between higher-order association areas. Using diffusion imaging, Catani *et al.* reveal associations between ASD and white matter abnormalities of the frontal lobe, and suggest that the condition is linked to aberrant developmental trajectories of frontal networks that persist in adulthood.

## Graphical abstract

**Figure:**
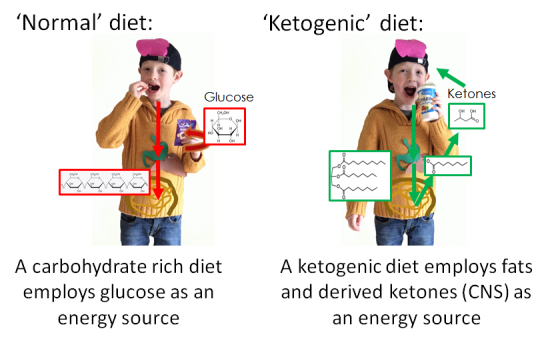
Autism spectrum disorder (ASD) is proposed to reflect atypical connectivity between higher-order association areas. Using diffusion imaging, Catani *et al.* reveal associations between ASD and white matter abnormalities of the frontal lobe, and suggest that the condition is linked to aberrant developmental trajectories of frontal networks that persist in adulthood.

## Introduction

Approximately 1% of the population have an autistic spectrum disorder (ASD), with a male:female ratio of 2.5:1 (Lai *et al.*, 2014). Recent advances in brain imaging have enabled *in vivo* mapping of the structural and functional characteristics associated with ASD. Several structural neuroimaging studies have implicated abnormalities in a number of brain regions, including the frontal and temporal cortex, the caudate nucleus, cerebellum, amygdala and hippocampus (Abell *et al.*, 1999; Waiter *et al.*, 2004; McAlonan *et al.*, 2005, 2008; Brieber *et al.*, 2007; Craig *et al.*, 2007; Bonilha *et al.*, 2008; Ke *et al.*, 2008; Lange *et al.*, 2010; Toal *et al.*, 2010; Cauda *et al.*, 2011; Via *et al.*, 2011; Nickl-Jockschat *et al.*, 2012; Nordahl *et al.*, 2012). Functional neuroimaging studies have also reported altered activation of these regions during tasks involving social, emotion and language processing (Baron-Cohen *et al.*, 1999; Pierce *et al.*, 2001; Just *et al.*, 2004; Ashwin *et al.*, 2007; Philip *et al.*, 2012). However, some of these findings have not been consistently replicated (Stigler *et al.*, 2011).

Inconsistency amongst previous findings is probably due to a variety of factors including small sample sizes, failure to apply strict diagnostic criteria and variability in confounding factors such as age and IQ (Freitag *et al.*, 2009). In addition, the majority of previous studies analysed only grey matter at the cortical and subcortical level. However, these regions do not function independently; rather they are interconnected through a complex system of short- and long-range tracts running within the white matter of each hemisphere (Catani and Bambini, 2014). White matter tract ‘connectivity’ regulates the speed and timing of activation across neural networks. These factors are necessary for optimum performance of higher-order tasks that rely on integrated information processing (Fields, 2008).

‘Atypical connectivity’ theories of ASD have postulated that higher-order association areas of the brain are partially disconnected during development (Belmonte *et al.*, 2004; Frith, 2004; Courchesne and Pierce, 2005; Geschwind and Levitt, 2007; Just *et al.*, 2012). Regional white matter structural abnormalities in ASD have been reported using different methods, from early region of interest approaches on structural MRI scans to, more recently, tract-specific virtual dissections of diffusion tensor MRI (DT-MRI) datasets (for a recent review see Ameis and Catani, 2015). Both approaches are mainly hypothesis driven and have therefore usually been limited to single regions or specific tracts (Aoki *et al.*, 2013), such as the midsagittal portion of the corpus callosum (Frazier and Hardan, 2009), or the core of the arcuate and uncinate fasciculus (Hardan *et al.*, 2000; Fletcher *et al.*, 2010), but sample sizes have tended to be small (Billeci *et al.*, 2012; Travers *et al.*, 2012; Mueller *et al.*, 2013). Other studies based on meta-analytical approaches (Radua *et al.*, 2011; Aoki *et al.*, 2013; Cauda *et al.*, 2014), voxel-based morphometry (Barnea-Goraly *et al.*, 2004; Keller *et al.*, 2007; Cheung *et al.*, 2009; Ke *et al.*, 2009; Lee *et al.*, 2009; Bloemen *et al.*, 2010; Noriuchi *et al.*, 2010; Groen *et al.*, 2011; Jou *et al.*, 2011), and tract-based spatial statistics (TBSS) (Barnea-Goraly *et al.*, 2010; Cheng *et al.*, 2010; Sahyoun *et al.*, 2010; Jou *et al.*, 2011; Shukla *et al.*, 2010; Walker *et al.*, 2012) have attempted to overcome some of the limitations attributable to small sample size and/or operator-dependent biases. However, it remains unclear whether the findings, if replicated in larger studies, are indicative of tract-specific anomalies in ASD or part of a more generalized brain abnormality (Wolff *et al.*, 2012). Therefore in this study we used two complementary approaches to analyse white matter connections in adults with ASD.

First, we applied TBSS (Smith *et al.*, 2006). This is a fully automated, operator-independent approach that permits a whole-brain analysis of white matter integrity in a voxel-wise manner. It has the potential to identify white matter differences in regions not previously considered to be of importance in a particular cohort and is resistant to operator bias. However, TBSS is affected by partial volume effects in regions accurate localization of between-group differences within specific tracts can be difficult with TBSS, as differences are often found in regions containing more than one tract (Catani, 2006). Therefore, after identifying regions of interest using TBSS, our second approach focused on specific tracts in greater detail using an approach based on DT-MRI tractography.

DT-MRI tractography facilitates the reconstruction of 3D trajectories of specific white matter tracts and probe their microstructural integrity. It permits a more detailed analysis of specific subpopulations of fibres, and indirect volumetric indices (e.g. number of streamlines and tract volume), which cannot be measured with TBSS. It has been applied to a wide range of neurodevelopmental conditions in addition to ASD (Catani *et al.*, 2011), including psychopathy (Craig *et al.*, 2009; Sethi *et al.*, 2015), dyslexia (Vanderauwera *et al.*, 2015), acallosal brain (Forkel *et al.*, 2014; Bénézit *et al.*, 2015) and schizophrenia (Kanaan *et al.*, 2009; Catani *et al.*, 2011). These studies suggest that this method of white matter sampling may be particularly well suited for neurodevelopmental conditions, including ASD, as changes are likely to occur along the entire course of the fibres, rather than be localized within circumscribed areas.

In summary, our aim was to understand whether white matter abnormalities in ASD are diffuse or localized to specific tracts. We also hypothesized that in adults with ASD the intensity of the white matter differences would be linked to the severity of symptoms in social interactions, repetitive behaviour and communication.

## Materials and methods

### Participants

Sixty-one male right-handed adults with ASD and 61 matched neurotypical male controls aged 18 to 45 years were recruited by advertisement and subsequently assessed at one of two collaborating autism research centres in the UK that make up the Medical Research Council UK Autism Imaging Multicentre Study (MRC AIMS) Consortium: the Institute of Psychiatry at Kings College London and the Autism Research Centre at the University of Cambridge. Equal ratios of cases to controls were recruited at each site: London, 34:34; Cambridge, 27:27. Overall intellectual ability was assessed using the Wechsler Abbreviated Scale of Intelligence (Wechsler, 1999). All participants fell within the high-functioning range on the spectrum defined by a full-scale IQ > 70. All participants with ASD were diagnosed according to International Statistical Classification of Diseases, 10th Revision research criteria (ICD10-R) (WHO, 1983), confirmed using the Autism Diagnostic Interview-Revised (ADI-R) (Lord *et al.*, 1994). The ADI-R is a semi-structured interview of parents that focuses primarily on the key diagnostic characteristics specified in the ICD10-R and DSM IV-R (Diagnostic and Statistical Manual of Mental Disorders); namely those features concerned with developmental delays, differences in reciprocal social interactions, language, communication and play, and restricted and stereotyped behaviours and interests. Many items concentrate on the age 4 to 5 year period.

All cases of ASD reached ADI-R algorithm cut-off values in the three domains of autism characteristics (social reciprocity, communication, repetitive and restricted behaviours and interests), although failure to reach cut-off in one of the domains by one point was permitted. Current symptoms were assessed using the Autism Diagnostic Observation Schedule (ADOS) (Lord *et al.*, 2000) but were not used as inclusion criteria. The ADOS is a semi-structured assessment of communication, social interaction and imaginative use of materials. Module 4 is designed for use with verbally fluent adolescents or adults, and describes a standardized interview/observational assessment consisting of 9–14 activities with 31 accompanying ratings. In addition, Autism-Spectrum Quotient (AQ), Empathy Quotient (EQ), and Systemizing Quotient (SQ) were collected for all patients. The ASD sample included 24 males with an ICD10-R diagnosis of childhood autism and 37 with Asperger syndrome (see Table 1 for details).

**Table 1:** Subject demographics

Characteristics^a^	Healthy controls (*n* = 61)	ASD group (*n* = 61)
Age, years	28 (±6.7) [18–45]	26 (±6.9) [18–41]
Full-scale IQ, WASI	114 (±11.1) [86–137]	111 (±13) [77–135]
Performance IQ, WASI^b^	116 (±10.5) [94–135]	109 (±14.9) [75–138]
Verbal IQ, WASI	110 (±12.5) [71–137]	110 (±13.2) [84–139]
EHI	96 (±7.6) [65–100]	93 (±14.2) [25–100]
ADI-R Score		
Social^c^	-	18.1 (±5.3)
Communication^c^	-	13.6 (±4.2)
Repetitive behaviour^c^	-	4.8 (±2.2)
ADOS Score		
Social^d^	-	6.1 (±2.9)
Communication^d^	-	3.3 (±1.7)
Repetitive behaviour^d^	-	1.2 (±1.2)
AQ	-	23.6 (±10.7)
EQ	-	32.7 (±15.5)
SQ	-	64.3 (±22.3)

Numbers are means ± standard deviations. Ranges are between square brackets. AQ = Autism-Spectrum Quotient; EQ = Empathy Quotient; SQ = Systemizing Quotient; WASI = Wechsler Abbreviated Scale of Intelligence.

^a^There were no significant differences between the ASD and control groups in age, full-scale IQ, or verbal IQ at *P* < 0.05 (two-tailed).

^b^There was a significant difference in performance IQ (*P* = 0.005).

^c^Information was available for all 61 individuals with ASD. The following cut-off scores were used: ADI-R Social, > 10; Communication, > 8; and Repetitive behaviour, >3.

^d^Information was available for 59 individuals with ASD. A cut-off of 7 was used for Communication plus Social interaction.

Exclusion criteria included a history of psychotic disorders, head injury, genetic disorder associated with ASD, any medical condition affecting brain function (e.g. epilepsy), current use of antipsychotic medication, mood stabilizers, or benzodiazepines or a history of substance misuse (including alcohol).

All participants gave informed written consent in accordance with ethics approval by the National Research Ethics Committee, Suffolk, England.

### MRI data acquisition

At both sites participants were scanned with MRI scanners operating at 3 T (GE Medical Systems HDx). We used the same scanning acquisition protocol at both sites to ensure data compatibility. High-resolution structural T_1_-weighted volumetric images were acquired with full-head coverage, 196 contiguous slices (1.1 mm thickness, with 1.09 × 1.09 mm in-plane resolution), a 256 × 256 × 196 matrix and a repetition time/echo time of 7/2.8 ms (flip angle 20°, field of view 28 cm). An 8-channel head-coil was used for radiofrequency transmission and reception allowing a parallel imaging (ASSET) speed up factor of 2. Consistent image quality was ensured by a semi-automated quality control procedure. DT-MRI data were acquired using a spin-echo echo-planar imaging double refocused sequence providing whole head coverage with isotropic image resolution (2.4 × 2.4 × 2.4 mm; 32 diffusion-weighted volumes with different non-collinear diffusion directions with b-factor 1300 s/mm^2^, and six non-diffusion-weighted volumes; 60 slices without slice gap; echo time = 104.5 ms; repetition time = 20 R-R intervals; 128 × 128 acquisition matrix; field of view 307 × 307 mm). The acquisition was gated to the cardiac cycle using a digital pulse oximeter placed on participants’ forefinger.

### Diffusion tensor MRI processing

Diffusion data were processed using ExploreDTI (Leemans *et al.*, 2009). Data were first preprocessed correcting for eddy current distortions and head motion. For each subject the mean rotational and translational relative movement was extracted and statistical analysis was performed to detect group differences in head movement. No statistically significant differences were found between ASD and controls. For each subject the b-matrix was then reoriented to provide a more accurate estimate of diffusion tensor orientations (Leemans and Jones, 2009). Diffusion tensor was estimated using a non-linear least square approach (Jones and Basser, 2004). Fractional anisotropy, mean diffusivity and radial diffusivity maps were generated. Whole brain tractography was performed by selecting all seed voxels with fractional anisotropy > 0.2. Streamlines were propagated using an Euler integration (Basser *et al.*, 2000), and a tractography algorithm step size of 1 mm. Where fractional anisotropy < 0.2 or when the angle between two consecutive tractography steps was > 35°, tractography stopped. Finally, diffusion tensor maps and whole brain tractography were exported to Trackvis (Wang *et al.*, 2007) for virtual manual dissection of the tracts.

### Tract-based spatial statistics analysis

Each participant’s fractional anisotropy map was transformed into standard stereotactic space (using FMRIB58 template) and a mean fractional anisotropy map for the whole sample used to create the average core ‘skeleton’. By using only the core of the sample’s white matter the peripheral tract regions are not involved in further analysis, thus removing partial volume effects (Smith *et al.*, 2006). Skeleton images of each participant’s fractional anisotropy map were then produced and projected onto the mean skeleton to identify voxels where fractional anisotropy value differs significantly between these skeletons using voxel-wise statistics. The design matrix used centre, performance IQ, and age as covariates. Five thousand permutations were applied (confidence limits for *P* = 0.05 is ± 0.0062). The TFCE value images are reported fully corrected for multiple comparisons across space using threshold free cluster enhancement with a specified significance level of *P* < 0.05.

### Tractography dissections

Following the TBSS analysis, white matter regions that were found to be atypical in ASD were localized using a probabilistic digital atlas of the major white matter tracts (Catani and Thiebaut de Schotten, 2012). Tracts that were identified to be atypical in ASD by the TBSS analysis were dissected using one or two region of interest approach (Catani and Thiebaut de Schotten, 2008). The tractographer was blind to group membership of each subject and brain side.

We performed virtual dissections of the left and right arcuate fasciculus and its segments connecting frontal, parietal and temporal regions according to previous publications (Catani *et al.*, 2005) (Fig. 2). We also performed virtual dissections of the three major limbic pathways including the cingulum, uncinate fasciculus and fornix (Catani and Thiebaut de Schotten, 2008). In addition we dissected the inferior longitudinal fasciculus and the inferior fronto-occipital fasciculus to extend the analysis to pathways that contain parts of the fibres connecting limbic structures to visual and auditory associative areas (Fig. 3). The corpus callosum was dissected using a single region of interest defined on the two most medial slices of the fractional anisotropy maps. Individual portions of the corpus callosum were analysed separately according to Witelson’s (1989) criteria.

### Statistical analysis

Statistical comparisons of the data were performed using SPSS software (SPSS Inc, Chicago, Ill). Demographic and behavioural differences between ASD and control groups were analysed using independent-samples *t*-tests. Repeated measures ANOVA analyses were performed separately for each measurement (i.e. number of streamlines, volume, fractional anisotropy, mean diffusivity, and perpendicular diffusivity) to examine group differences (i.e. the fixed factor) for the perisylvian network (long, posterior and anterior segments of the arcuate fasciculus) and the limbic association pathways (cingulum and uncinate fasciculus). Corpus callosum’s group differences were estimated separately for each measurement (i.e. number of streamlines, volume, fractional anisotropy, mean diffusivity, and perpendicular diffusivity) using univariate ANOVA analyses. Age, centre and performance IQ were entered as covariates in all analyses. *Post hoc* comparisons for specific tracts were considered as statistically significant if they survived Bonferroni correction for multiple comparisons (*P* < 0.0045, five tracts for each hemisphere; *P* < 0.008 for the corpus callosum). Both streamline count and tract volume were calculated for each tract. Considering that both measures yielded to similar results only streamline count is reported. To study possible associations between tract abnormalities and symptom severity within the ASD group we related differences in tracts to symptom severity as measured by the relevant ADI-R and ADOS scores using Pearson correlation.

## Results

### Tract-based spatial statistics

**Figure 1:**
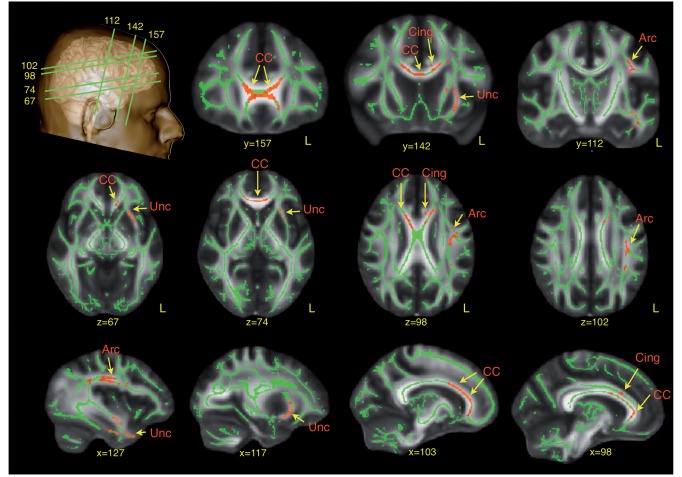
**TBSS analysis of differences in fractional anisotropy between ASD subjects and neurotypical controls.** Red regions indicate reduced fractional anisotropy values in ASD. Arc = arcuate fasciculus; CC = corpus callosum; Cing = cingulum; Unc = uncinate fasciculus.

Compared to controls, the ASD group had significantly (corrected for multiple comparisons) lower fractional anisotropy in the left arcuate fasciculus (*P* = 0.026), external capsule (*P* = 0.046), anterior (*P* = 0.044) and posterior (*P* = 0.032) cingulum, and anterior corpus callosum (*P* = 0.015). There were no increases in fractional anisotropy in the ASD group compared to controls (Fig. 1).

### Diffusion tractography analysis

#### Arcuate fasciculus

Between-groups differences were statistically significant for the mean diffusivity [*F*(1 120) = 5.618; *P* = 0.020] and radial diffusivity [*F*(1 120), 4.629; *P* = 0.034]. A statistically significant Group × Hemisphere interaction for the number of streamlines [*F*(2 116) = 6.817; *P* = 0.010] and Group × Tract interaction for the number of streamlines [*F*(2, 113) = 4.045; *P* = 0.02] was also found.

*Post hoc* independent sample *t*-test, Bonferroni corrected for multiple comparisons, showed that the ASD group had higher mean and radial diffusivity in the left long and left anterior segments (Table 2). Differences in the number of streamlines of the left long and anterior segments and mean diffusivity of the posterior right segment did not survive correction for multiple comparisons.

**Table 2:** Tract-specific measurements of the three segments of the arcuate fasciculus

Segments	Measurements	Controls	ASD	*P*-value
**Long left**	Streamlines	292 ± 108	246 ± 108	0.023
	FA	0.504 ± 0.020	0.496 ± 0.023	0.042
	MD	0.74 ± 0.02 × 10 ^− 3^	0.75 ± 0.02 × 10^−3^	0.002*
	RD	0.51 ± 0.02 × 10^−3^	0.52 ± 0.02 × 10^−3^	0.003*
**Long right**	Streamlines	153 ± 116	120 ± 88	0.084
	FA	0.480 ± 0.024	0.470 ± 0.025	0.21
	MD	0.49 ± 0.02 × 10^−3^	0.48 ± 0.06 × 10^−3^	0.163
	RD	0.52 ± 0.02 × 10^−3^	0.52 ± 0.02 × 10^−3^	0.09
**Anterior left**	Streamlines	132 ± 78	101 ± 63	0.018
	FA	0.459 ± 0.026	0.448 ± 0.030	0.039
	MD	0.75 ± 0.02 × 10^−3^	0.77 ± 0.03 × 10^−3^	0.003*
	RD	0.55 ± 0.02 × 10^−3^	0.57 ± 0.03 × 10^−3^	0.003*
**Anterior right**	Streamlines	210 ± 125	192 ± 119	0.431
	FA	0.470 ± 0.027	0.464 ± 0.024	0.188
	MD	0.76 ± 0.02 × 10^−3^	0.77 ± 0.02 × 10^−3^	0.083
	RD	0.55 ± 0.02 × 10^−3^	0.55 ± 0.02 × 10^−3^	0.078
**Posterior left**	Streamlines	156 ± 67	170 ± 94	0.353
	FA	0.454 ± 0.026	0.453 ± 0.023	0.885
	MD	0.75 ± 0.02 × 10^−3^	0.76 ± 0.02 × 10^−3^	0.007
	RD	0.54 ± 0.02 × 10^−3^	0.55 ± 0.03 × 10^−3^	0.057
**Posterior right**	Streamlines	132 ± 75	131 ± 83	0.947
	FA	0.452 ± 0.028	0.451 ± 0.026	0.878
	MD	0.76 ± 0.02 × 10^−3^	0.77 ± 0.03 × 10^−3^	0.011
	RD	0.55 ± 0.02 × 10^−3^	0.56 ± 0.03 × 10^−3^	0.055

Numbers are means ± SD. *Indicates values that survive Bonferroni correction for multiple comparisons. FA = fractional anisotropy; MD = mean diffusivity; RD = radial diffusivity.

**Figure 2:**
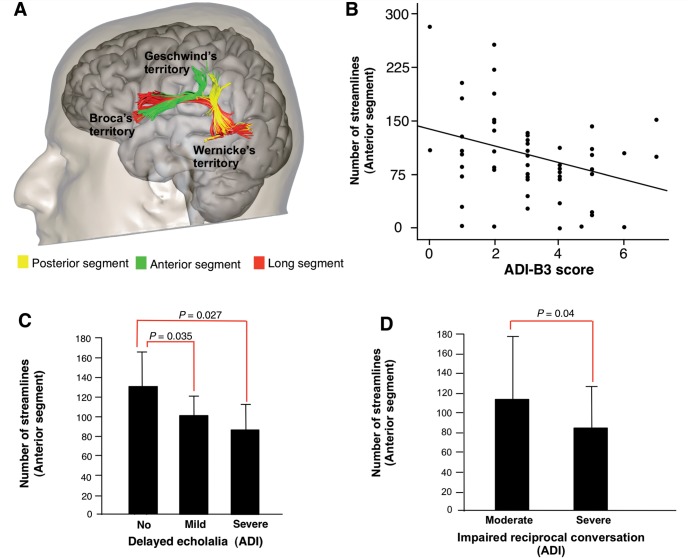
**The anatomy of the arcuate fasciculus in relation to childhood ASD symptoms.** (**A**) Tractography dissections of the three segments of the arcuate fasciculus. (**B**) Negative correlation between the number of streamlines of the left anterior segment and the B3 score (stereotyped, repetitive and idiosyncratic speech) of the ADI-R (r = −0.340; *P* = 0.005) in the ASD group. (**C**) In the ASD group, participants with a significant history of stereotyped utterance and delayed echolalia in childhood had a significantly lower number of streamlines in the anterior segment of the arcuate fasciculus. (**D**) Similarly, ASD subjects with severely impaired reciprocal conversation in childhood had a significantly lower number of streamlines in the anterior segment of the arcuate fasciculus compared to ASD subjects with moderate symptoms.

We subsequently explored whether anatomical differences in the left long and anterior segments of the arcuate fasciculus were associated with language and communication impairment. Pearson’s correlations were calculated between tract-specific measurements and measures for abnormalities in language and communication, that is, ADI-R (B1-B4 subscores) and ADOS (A subscore) subscores in the ‘communication’ domain. A statistically significant negative correlation was found between the number of streamlines of the left anterior segment and the ADI-R B3 score for stereotyped, repetitive and idiosyncratic speech (Pearson’s correlation = −0.340; *P* = 0.005) (Fig. 2B). The correlation was mainly driven by the severity of stereotyped utterances and delayed echolalia (Fig. 2C) and impaired reciprocal conversation (Fig. 2D). There were no significant correlations with the ADOS communication scores.

#### Limbic tracts

A statistically significant Group × Hemisphere interaction for fractional anisotropy [*F*(2 118) = 6.197; *P* = 0.014] was found. No other interactions were found with number of streamlines, mean diffusivity or radial diffusivity.

*Post hoc* independent sample *t*-test, Bonferroni corrected for multiple comparisons, showed that the ASD group had significantly higher mean diffusivity and radial diffusivity in the left uncinate and lower fractional anisotropy in the left cingulum (Table 3). Differences in the number of streamlines and fractional anisotropy of the left uncinate and radial diffusivity in the left cingulum did not survive correction for multiple comparisons.

**Table 3:** Tract-specific measurements of the limbic tracts

Limbic tracts	Measurements	Controls	ASD	*P*-value
**Uncinate left**	Streamlines	180 ± 80	147 ± 68	0.019
	FA	0.462 ± 0.022	0.452 ± 0.027	0.023
	MD	0.82 ± 0.02 × 10^−3^	0.84 ± 0.02 × 10^−3^	0.003*
	RD	0.59 ± 0.03 × 10^−3^	0.61 ± 0.03 × 10^−3^	0.001*
**Uncinate right**	Streamlines	206 ± 81	183 ± 78	0.149
	FA	0.468 ± 0.022	0.459 ± 0.029	0.044
	MD	0.82 ± 0.02 × 10 ^− 3^	0.83±0.40×10 ^− 3^	0.014
	RD	0.58 ± 0.02 × 10^−3^	0.60 ± 0.04 × 10^−3^	0.025
**Cingulum left**	Streamlines	666 ± 139	607 ± 149	0.036
	FA	0.479 ± 0.025	0.466 ± 0.023	0.003*
	MD	0.78 ± 0.02 × 10^−3^	0.79 ± 0.02 × 10^−3^	0.199
	RD	0.55 ± 0.02 × 10^−3^	0.56 ± 0.02 × 10^−3^	0.007
**Cingulum right**	Streamlines	596 ± 126	562 ± 137	0.158
	FA	0.464 ± 0.024	0.457 ± 0.025	0.083
	MD	0.78 ± 0.02 × 10 ^− 3^	0.79 ± 0.02 × 10^−3^	0.147
	RD	0.56 ± 0.02 × 10^−3^	0.58 ± 0.03 × 10^−3^	0.064
**IFOF left**	Streamlines	182 ± 80	167 ± 88	0.481
	FA	0.499 ± 0.025	0.493 ± 0.025	0.272
	MD	0.78 ± 0.02 × 10 ^− 3^	0.80 ± 0.03 × 10 ^− 3^	0.008
	RD	0.54 ± 0.03 × 10 ^− 3^	0.55 ± 0.03 × 10 ^− 3^	0.025
**IFOF right**	Streamlines	166 ± 81	177 ± 93	0.35
	FA	0.488 ± 0.020	0.491 ± 0.025	0.5
	MD	0.79 ± 0.02 × 10 ^− 3^	0.80 ± 0.03 × 10 ^− 3^	0.023
	RD	0.55 ± 0.02 × 10 ^− 3^	0.56 ± 0.03 × 10 ^− 3^	0.277
**ILF left**	Streamlines	235 ± 74	222 ± 118	0.469
	FA	0.529 ± 0.027	0.524 ± 0.023	0.138
	MD	0.79 ± 0.03 × 10 ^− 3^	0.81 ± 0.03 × 10 ^− 3^	0.035
	RD	0.53 ± 0.02 × 10 ^− 3^	0.54 ± 0.03 × 10 ^− 3^	0.024
**ILF right**	Streamlines	265 ± 103	228 ± 93	0.021
	FA	0.516 ± 0.070	0.524 ± 0.021	0.894
	MD	0.80 ± 0.02 × 10 ^− 3^	0.81 ± 0.02 × 10 ^− 3^	0.02
	RD	0.53 ± 0.02 × 10 ^− 3^	0.54 ± 0.03 × 10 ^− 3^	0.086

Numbers are means ± SD. *Indicates values that survive Bonferroni correction for multiple comparisons. FA = fractional anisotropy; MD = mean diffusivity; RD = radial diffusivity; IFOF = inferior fronto-occipital fasciculus; ILF = inferior longitudinal fasciculus.

**Table 4:** Tract-specific measurements of the callosal tracts

Callosal segments	Measurements	Controls	ASD	*P*-value
**Genu**	Streamlines	914 ± 304	853 ± 192	0.197
	FA	0.572 ± 0.022	0.566 ± 0.020	0.178
	MD	0.82 ± 0.04 × 10 ^− 3^	0.84 ± 0.03 × 10 ^− 3^	0.006*
	RD	0.51 ± 0.03 × 10 ^− 3^	0.53 ± 0.03 × 10 ^− 3^	0.011
**Anterior body**	Streamlines	412 ± 156	427 ± 143	0.534
	FA	0.539 ± 0.022	0.532 ± 0.018	0.062
	MD	0.82 ± 0.03 × 10 ^− 3^	0.83 ± 0.03 × 10 ^− 3^	0.003*
	RD	0.53 ± 0.03 × 10 ^− 3^	0.55 ± 0.05 × 10 ^− 3^	0.004*
**Centre body**	Streamlines	460 ± 149	427 ± 111	0.191
	FA	0.558 ± 0.022	0.550 ± 0.020	0.04
	MD	0.81 ± 0.03 × 10 ^− 3^	0.82 ± 0.03 × 10 ^− 3^	0.04
	RD	0.51 ± 0.03 × 10 ^− 3^	0.52 ± 0.03 × 10 ^− 3^	0.022
**Posterior body**	Streamlines	464 ± 120	463 ± 118	0.98
	FA	0.572 ± 0.022	0.574 ± 0.018	0.537
	MD	0.79 ± 0.03 × 10 ^− 3^	0.79 ± 0.02 × 10 ^− 3^	0.741
	RD	0.49 ± 0.03 × 10 ^− 3^	0.49 ± 0.02 × 10 ^− 3^	0.994
**Isthmus**	Streamlines	474 ± 143	453 ± 131	0.363
	FA	0.559 ± 0.024	0.557 ± 0.020	0.723
	MD	0.80 ± 0.03 × 10 ^− 3^	0.79 ± 0.02 × 10 ^− 3^	0.532
	RD	0.50 ± 0.03 × 10 ^− 3^	0.50 ± 0.02 × 10 ^− 3^	0.722
**Splenium**	Streamlines	1514 ± 295	1498 ± 315	0.774
	FA	0.611 ± 0.017	0.608 ± 0.018	0.424
	MD	0.79 ± 0.02 × 10 ^− 3^	0.79 ± 0.02 × 10 ^− 3^	0.584
	RD	0.46 ± 0.02 × 10 ^− 3^	0.47 ± 0.02 × 10 ^− 3^	0.44

Numbers are means ± SD. *Indicates values that survive Bonferroni correction for multiple comparisons. FA = fractional anisotropy; MD = mean diffusivity; RD = radial diffusivity.

**Figure 3:**
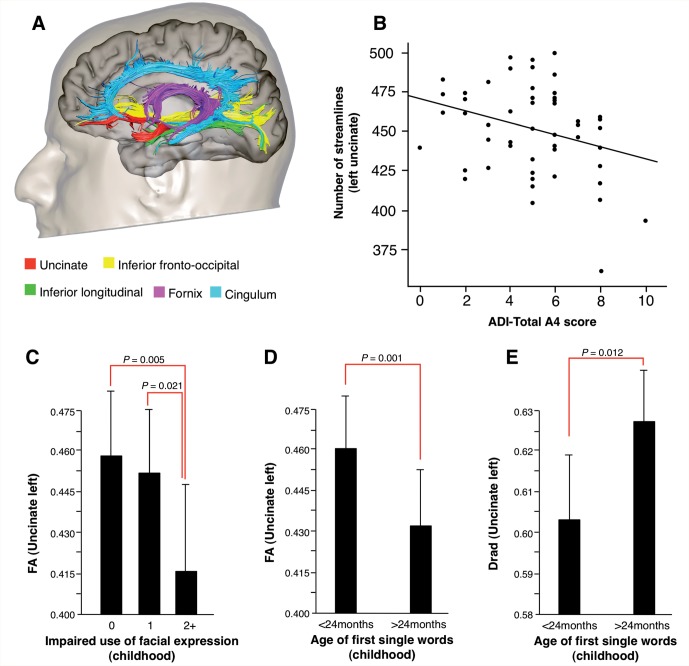
**The anatomy of the limbic tracts in relation to childhood ASD symptoms.** (**A**) Tractography reconstructions of the limbic pathways. (**B**) Negative correlation between the number of streamlines of the left uncinate fasciculus and the total A4 score for impaired socioemotional reciprocity in the ADI-R (Pearson’s correlation = −0.295; *P* = 0.01) in the ASD group. ASD participants with a (**C**) significant history of impaired use of facial expression in childhood and (**D** and **E**) late use of first single words had a significantly lower fractional anisotropy (FA) and higher radial diffusivity in the left uncinate fasciculus.

We also explored whether anatomical differences in the left uncinate fasciculus and cingulum were associated with impaired social interaction. Pearson’s correlation was calculated between tract-specific measurements and measures for abnormality in social interaction, that is, ADI-R (A1–A4 subscores) and ADOS (B subscore). A statistically significant correlation was found between the number of streamlines of the left uncinate and the ADI-R total score for qualitative abnormalities in reciprocal social interactions (r = −0.269; *P* = 0.01), and A4 score for impaired socio-emotional reciprocity (r = −0.295; *P* = 0.01) (Fig. 3B). A similar correlation was found between fractional anisotropy of the left uncinate and the A4 score (r = −0.301; *P* < 0.01). The correlation with A4 score was mainly driven by the severity of ‘inappropriate use of facial expression’ (Fig. 3C). Statistically significant differences in the fractional anisotropy and radial diffusivity of the left uncinate fasciculus were also found between ASD participants who demonstrated delayed use of meaningful single words (<24 months of age) and ASD participants who developed use of single words within normal developmental ranges (Fig. 3D and E). There were no correlations between ADOS subscores and uncinate measurements, or between scores for impaired socio-emotional reciprocity on the ADI-R and ADOS and other limbic tracts.

**Figure 4:**
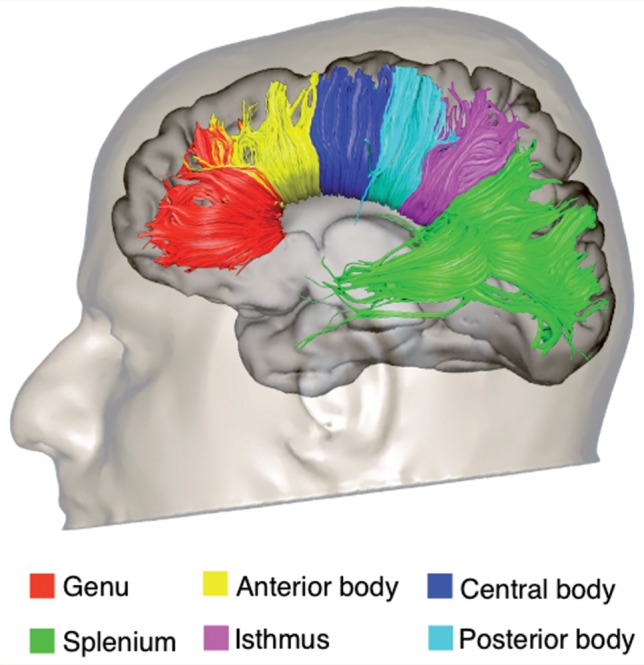
**Tractography reconstruction of the segments of the corpus callosum according to Witelson’s division (1989)**.

#### Corpus callosum

There were no statistically significant differences between participants with ASD and controls in the number of streamlines, fractional anisotropy, mean diffusivity and radial diffusivity of the total corpus callosum.

The analysis of the segments of the corpus callosum revealed a statistically significant increase in mean diffusivity and radial diffusivity in the genu and anterior body in the ASD group as compared to controls. Differences in fractional anisotropy and radial diffusivity of the central body did not survive correction for multiple comparisons. No statistically significant differences were found in the posterior body, isthmus and splenium. There were no significant correlations between diffusion indices of the corpus callosum and symptom severity as measured by the ADI-R and ADOS.

## Discussion

In this multicentre study we investigated the anatomy of white matter networks in adults with ASD. We were able to confirm in a large population that white matter differences in ASD persist into adult life. White matter differences in ASD were localized to major association and commissural tracts of the frontal lobe. These tracts connect frontal lobe to more posterior areas of the parietal, limbic and temporal lobe. Further, white matter differences within particular networks were found to be associated with specific childhood ASD behavioural characteristics. We found, for example, differences in the left arcuate fasciculus, which plays a key role in language and social function (Catani and Bambini, 2014). Specifically, the number of streamlines of the left anterior segment of the arcuate fasciculus was negatively associated with stereotyped utterances and delayed echolalia in childhood (i.e. based on the ADI-R). Similarly, we found that ASD was associated with diffusion abnormalities of the left uncinate fasciculus, which plays a significant role in face encoding and emotional processing associated with face perception (Papagno *et al.*, 2011). Further, this correlated with ‘inappropriate use of facial expression’ in childhood.

Overall, our findings confirm a number of previous studies that have examined white matter tracts in ASD with varying approaches to diffusion imaging data (see Ameis and Catani, 2015 for a recent review). A recent meta-analysis of diffusion tensor imaging (voxel-based) studies that provided a collective sample of 330 people with ASD and 313 controls revealed decreases in fractional anisotropy in white matter voxels containing fibres of the left arcuate fasciculus, uncinate fasciculus and corpus callosum, and increases in mean diffusivity in the same regions (Aoki *et al.*, 2013). The meta-analysis also reported differences in the inferior longitudinal and inferior fronto-occipital fasciculus, but these did not reach statistical significance. In addition to voxel-based studies, tractography studies have also examined white matter tracts linking socio-emotional and communication regions in ASD. While the uncinate and arcuate fasciculi have been most frequently implicated as abnormal in ASD, a smaller number of studies support the presence of abnormalities in additional tracts, including the cingulum bundle, the inferior longitudinal and inferior fronto-occipital fasciculus (Ameis and Catani, 2015). Such abnormalities are generally reflected by decreased fractional anisotropy and increased diffusivity, although differences in the opposite direction have also been reported in children and adolescents, but less frequently (Cheng *et al.*, 2010; Shukla *et al.*, 2010). Some studies have also reported increased number of streamlines for the same tracts (Pugliese *et al.*, 2009; Kumar *et al.*, 2010; Thomas *et al.*, 2011). While small sample size, differences in the severity of the disorder and in the imaging approaches among studies can explain some of the contrasting findings (Lazar *et al.* 2014; Ameis and Catani, 2015), different diffusion abnormalities in the same tract in studies sampling ASD participants of different ages, likely point to an evolving picture of white matter pathology with development (Pugliese *et al.*, 2009; Kleinhans *et al.*, 2012).

An unexpected finding was the absence of significant correlations between tract-specific measurements and severity of clinical symptoms in adulthood as measured by the ADOS. One possible explanation for this negative finding is that ADOS scores, although commonly used to support a diagnosis of ASD, may not be suitable for quantifying the exact severity of current symptoms, especially in adults. It is also possible that participants with ASD compensated for their childhood deficits through observation of other people and alternative modes of communication. This has been widely documented for the language deficits by previous behavioural studies (Eisenmajer *et al.*, 1996; Gilchrist *et al.*, 2001; Howlin *et al.*, 2003). This may lead to changes at the functional, or cortical level, but not within the white matter—leading to a mismatch between white matter abnormalities and current symptoms severity. Indeed, in a recent study we showed that in ASD, current and historical language symptoms were associated with different neuroanatomical correlates (Lai *et al.*, 2014). In general there is limited evidence in the literature of a direct correlation between white matter abnormalities and clinical symptoms. The most common finding reported is a direct correlation between uncinate fasciculus abnormalities and impaired social cognition. For example, Chen *et al.* (2011) reported a negative correlation between white matter indices and social deficits in ASD, where increased Social Responsiveness Scale scores correlated with reduced fractional anisotropy in right uncinate fasciculus. Similarly Kumar et al. (2010) used tractography to show that macrostructural alterations within the uncinate fasciculus in ASD were correlated with the severity of symptoms, including socio-emotional deficits, on the Gilliam Autism Rating Scale. One interesting study that used DTI after a communication intervention in 22 low functioning young males with ASD found a positive correlation of uncinate fasciculus fractional anisotropy with both therapy duration and symptom improvement measured using the Child Autism Rating Scale (Pardini *et al.*, 2012). While all of these findings suggest that ASD is characterized by differences in white matter tracts, the exact biological mechanism modulating these differences cannot be elucidated by diffusion techniques but may be understood by other methodologies, including post-mortem studies.

Post-mortem studies have reported that ASD is associated with white matter inflammation and reduced neural size and increased packing density within frontal and temporal limbic (grey matter) structures (Bailey *et al.*, 1998; Bauman and Kemper, 2005; Vargas *et al.*, 2005). Further, compared to controls, ASD is associated with increased number of neuronal processing units, known as cortical mini-columns (Mountcastle, 1997), within frontal and temporal cortices (Casanova *et al.*, 2002*a*, *b*). Mini-columns are highly interconnected, and a greater number of mini-columns could result in increased formation of short-range (intracortical) connections and disrupted maturation of long-range connections linking distant regions (Casanova *et al.*, 2002*a*, *b*). These findings may be of particular relevance to our study as they suggest ASD pathology induces white matter changes in the long white matter connections possibly related to axons with larger diameter, reduced myelination, or increased oedema due to inflammation (Vargas *et al.*, 2005). This interpretation could explain the findings of reduced fractional anisotropy and increased radial diffusivity in the long-range connections of the frontal lobe in the current study. This hypothesis is also supported by a recent post-mortem study in ASD, which reported reduced myelin thickness in the frontal white matter (Zikopoulos and Barbas, 2010).

It remains unclear, however, whether these changes follow an initial failure of connections to establish correct organization, or a later weakening of successfully formed white matter connections (Geschwind and Levitt, 2007) as the majority of ASD individuals studied in post-mortem analyses are older children and adults (Courchesne *et al.*, 2007). Therefore, to determine how, and when, these putative changes commence future studies would benefit from including a younger cohort. A recent *in vivo* longitudinal study has highlighted the need for this (Wolff *et al.*, 2012). In this study high-risk infants who later developed ASD were found to have reduced fractional anisotropy in limbic and association tracts including those reported in our current study, by the age they showed obvious signs of autism. This study also suggested that white matter differences are evident as early as 6 months of age in infants at risk of ASD, although abnormalities were in the opposite direction (i.e. increased fractional anisotropy in the at risk group). The initial increased fractional anisotropy could have been related to increased myelination, and/or smaller axonal diameter, in infants at risk of ASD. These findings suggest that ASD progresses through different stages during early neurodevelopment (i.e. overgrowth followed by blunted development). Realistically, it is difficult to validate these findings using post-mortem studies due to limited availability of donated brains from infants. However, the application of complimentary *in vivo* neuroimaging methods could assist in the interpretation of diffusion tensor imaging results.

Our findings also indicate an asymmetry of abnormalities with greater differences in the white matter of the left hemisphere in the ASD compared to controls. Previous diffusion studies have demonstrated that maturation of left white matter pathways in the temporal and frontal lobe is correlated with development of specific cognitive functions (Nagy *et al.*, 2004). Loss of normal interhemispheric asymmetry, both at the cortical and subcortical level, is one of the most replicated findings in ASD (Herbert *et al.*, 2005; Radua *et al.*, 2011; Ameis and Catani, 2015). This indicates that in general the underlying pathological process is rather asymmetrical in ASD and specific to those networks that show the greatest degree of lateralization in the neurotypically developing brain.

Finally, the differences in tract-specific measurements between neurotypical adults and ASD were particularly significant for the mean diffusivity and radial diffusivity in our tractography analysis. This finding suggests that, compared to fractional anisotropy and tract volume, mean diffusivity and radial diffusivity have greater intrinsic sensitivity to *in vivo* abnormalities associated with ASD pathology but also that the biophysical meaning of these indices is different. Since fractional anisotropy is a measure of anisotropy, it is by definition a ‘relative’ index that quantifies the difference of diffusivities of the three eigenvalues. This makes this index sensitive to changes in the overall structural organization of the tissue or the fibre architecture but it does not give information on how much faster or slower is diffusion in different directions. Conversely, mean diffusivity and radial diffusivity are absolute measures that directly provide a quantitative measure of water mobility within the tissue, either as average to the voxel (mean diffusivity) or radially to the direction of maximum diffusivity (radial diffusivity). Therefore mean diffusivity and radial diffusivity could be more specific and better related to abnormalities, such as changes in axonal membrane, extra-axonal volume density or in the myelin sheet because they may capture something not visible in a ratio or a difference in diffusivities. In support of our findings, a recent study also reported differences between typically developing adolescents and individuals with ASD only for diffusivity measures (Lisiecka *et al.*, 2015).

While the present study has several advantages compared to previous ones, its limitations include a cross-sectional design and exclusion of children, females and participants with intellectual disability, which limit our capability for generalization or confirming causal and temporal developmental effects. These criteria were implemented in order to optimize sample homogeneity. There is, for example, sex difference in arcuate tract development in the typically developing population (Catani *et al.*, 2007). Therefore our results may not generalize to females with ASD, suggesting the need for future studies to include comparable samples of females. Similarly, the exclusion of individuals with below average IQ may mean that our results are not generalizable to the proportion of those with both ASD and intellectual disability. It could be argued that the significant difference in the performance IQ between the two groups may have confounded our findings. However, this difference was dealt with by co-varying for this across all of our analyses. A multicentre design was used for MRI data acquisition, which may carry additional limitations. However, we used the same scanner model and acquisition parameters across scanning sites. In addition intersite effects were accounted for in the statistical model (Suckling *et al.*, 2010, 2012). Therefore, the detected between-group differences cannot be fully explained by these limitations. Finally, some of the negative findings (e.g. lack of correlation between anatomy of the corpus callosum and ASD symptoms) could be explained by the use of the tensor method, which is limited in resolving fibre crossing. Future studies are necessary to improve the complete visualization of callosal tracts using advanced diffusion models (Dell’Acqua *et al.*, 2013).

In conclusion, our results suggest that male adults with ASD have regional differences in brain anatomy, which persist in adult life and correlate with specific aspects of autistic symptoms in childhood . We also found that ASD was associated with specific structural abnormalities of white matter fibres, compatible with the concept of autism being associated with atypical developmental connectivity of the frontal lobes. Future studies are needed to confirm our findings and to extend them across a wider range of autistic spectrum conditions.

## Abbreviations

ADI-R: Autism Diagnostic Interview–Revised
ADOS: Autism Diagnostic Observation Schedule
ASD: autism spectrum disorder
TBSS: tract-based spatial statistics

## Appendix 1

The MRC AIMS Consortium is a UK collaboration of autism research centres in the UK including the Institute of Psychiatry, London, the Autism Research Centre, University of Cambridge, and the Autism Research Group, University of Oxford. It is funded by the MRC UK and headed by the Section of Brain Maturation, Institute of Psychiatry. The Consortium members are in alphabetical order:

Anthony J. Bailey, Simon Baron-Cohen, Patrick F. Bolton, Edward T. Bullmore, Sarah Carrington, Bhismadev Chakrabarti, Eileen M. Daly, Sean C. Deoni, Christine Ecker, Francesca Happé, Julian Henty, Peter Jezzard, Patrick Johnston, Derek K. Jones, Meng-Chuan Lai, Michael V. Lombardo, Anya Madden, Diane Mullins, Clodagh M. Murphy, Declan G. M. Murphy, Greg Pasco, Amber N. V. Ruigrok, Susan A. Sadek, Debbie Spain, Rose Stewart, John Suckling, Sally J. Wheelwright and Steven C. Williams.
